# Review of Dr. McElroy’s Hypothesis of the Pathology of Typhoid Fever

**Published:** 1868-05

**Authors:** J. E. Hendricks

**Affiliations:** Des Moines, Iowa


ARTICLE XVI.

### REVIEW OF DR. McELROY'S HYPOTHESIS OF THE PATHOLOGY OF TYPHOID FEVER.

By J. E. HENDRICKS, M.D., Des Moines, Iowa.

After describing the characteristic symptoms of typhoid fever, the writer says: "Now what is the meaning of this series of phenomena? * * * Manifestly, the soft tissues of the body are burning up. And why? Because the fever poison has rendered more or less of them unsuitable for further use of the body."

This assertion that the fever poison has rendered more or less of the soft tissues unsuitable for the use of the body, is, I think, an *entirely gratuitous assertion*, not supported by a single fact of observation or analogy. The hypothesis is not only not supported by observed facts or analogy, but it is directly opposed to *known* facts. As I take no exceptions to the treatment advocated, it being, in the main, the *expectant*, which I believe most experienced physicians adopt, I will confine these remarks to the above hypothesis.

Without expressing any opinion as to the nature of the *vital force*, or, as it is more properly called, the "*directing energy*," I may safely say that physiologists now agree that all the forces manifested by the animal system are ultimately derived from without, and are introduced as food. This food consists of molecules of matter in a state of unstable equilibrium, and force is only manifested when these molecules descend to a state of more stable equilibrium; and when the molecules thus descend to a more stable state, force, in some form, is *always* produced.

All the tissues, therefore, that are susceptible of oxidation are, until chemically decomposed, in a state of *potential* force, and cannot (unless cut off from a supply of oxygen) become "effete" or prejudicial to the system. That the soft tissues are not cut off from a supply of oxygen during fever, is manifest from the fact that they are being "burned up," producing, instead of muscular power, its equivalent, in the form of local *heat*, thereby clearly indicating that the tissue before its oxidation was *not* "effete," but in its normal state of *potential* force. In view of these facts (which are not mere hypotheses, but rest upon the firm basis of the conservation of forces), what is "effete tissue," which needs to be burned up that it may be eliminated? Manifestly, an *imaginary* source of disturbance, the existence of which is incompatible with the known facts.

But, it may be asked, if I deny that "effete" tissue is the proximate cause of fever, what *is* the cause?

As it is admitted that all the forces manifested by the system are ultimately derived from without, we may, for the sake of illustration, compare the system to a very complicated machine, the steam engine for instance. In the absence of any positive knowledge of the cause of fever, I will assume, as is generally believed, that the fever poison is some foreign body that is introduced and interferes with the harmonious action of the system; as if, instead of lubricating some of the moving parts of a steam engine with oil, some rough, adhesive substance should be applied; obviously, a portion of the working force of the engine would be diverted from its ordinary application, to overcome this adhesion or the friction resulting therefrom, and would manifest itself in the form of *heat*. So in the case of the fever patient; a poison has been introduced which interferes with the harmonious action of the different parts of the system; the assimilation of food is arrested; the force resulting from the oxidation of the tissues is directed from its usual manifestation as muscular power, and appears merely as an equivalent in the form of *heat*. This condition continues until the system eliminates the poison or becomes able to perform its wonted functions, notwithstanding its presence. There is, therefore, no necessity for assuming the presence of "effete" tissue, even if its existence were possible.

It may not be improper to add to the foregoing remarks, the following quotation from Dr. Carpenter:

"To sum up: The life of man or of any of the higher animals, essentially consists in the manifestation of forces of various kinds, of which the organism is the instrument; and these forces are developed by the retrograde metamorphosis of the organic compounds generated by the instrumentality of the plant, whereby they ultimately return to the simple binary forms (water, carbonic acid, and ammonia), which serve as the essential food of vegetables. Of these organic compounds, one portion (*a*) is converted into the substance of the living body by a constructive force which (in so far as it is not supplied by the direct agency of external heat) is developed by the retrograde metamorphosis of another portion (*b*) of the food. And whilst the ultimate descent of the first-named portion (*a*) to the simple condition from which it was originally drawn becomes one source of the peculiarly animal powers—the *psychical* and *motor*—exerted by the organism, another source of these may be found in a like metamorphosis of a further portion (*c*) of the food which has never been converted into living tissue."

